# Acute Toxicity Evaluation, Antibacterial, Antioxidant and Immunomodulatory Effects of *Melastoma malabathricum*

**DOI:** 10.3390/molecules17033547

**Published:** 2012-03-20

**Authors:** Zahra A. Amin Alnajar, Mahmood A. Abdulla, Hapipah M. Ali, Mohammed A. Alshawsh, A. Hamid A. Hadi

**Affiliations:** 1 Department of Molecular Medicine, Faculty of Medicine, University of Malaya, 50603, Kuala Lumpur, Malaysia; Email: ammeen@um.edu.my (M.A.A.); alshawshmam@yahoo.com (M.A.A.); 2 Department of Chemistry, Faculty of Science, University of Malaya, 50603, Kuala Lumpur, Malaysia; Email: hapipah@um.edu.my (H.M.A.); ahamid@um.edu.my (A.H.A.H.)

**Keywords:** *Melastoma malabathricum*, antioxidant, immunomodulatory, antibacterial, acute toxicity

## Abstract

*Melastoma malabathricum* (MM) is a well-known plant in Malaysian traditional medicine, locally known as senduduk. Its ethanol and aqueous extracts have been used in the present investigation to study the immunomodulatory role on human peripheral blood mononuclear cell (PBMC), and the DPPH, ABTS and FRAP free radical scavenging activities were also measured. Total flavonoids and total phenolic contents were assayed and the antibacterial effect was tested against four species of bacteria; two Gram-positive (*Staphylococcus aureus* and *Streptococcus agalactiae*) and two Gram-negative (*Escherichia coli* and *Klebsilla pneumonia*). The tests were carried out using the disc diffusion, minimum inhibitory concentration (MIC) and minimum bactericidal concentration (MBC) methods. Moreover, the acute toxicity was evaluated *in vivo* on the ethanol extract of MM to establish its safety when administered orally. In our results, both extracts of MM showed abilities to scavenge DPPH and ABTS free radicals, IC_50_ values: (11.599 ± 0.84, 10.573 ± 0.58 µmol/L) and (62.657 ± 0.78, 63.939 ± 0.48 µmol/L) for ethanol and aqueous extracts respectively. Indeed the ethanol extract evidenced high phenolic content (384.33 ± 0.005 mg/g), flavonoids contents (85.8 ± 0.009 mg/g) and ferric reducing antioxidant power (33,590 ± 0.038 mmol/g), with high activity against *S. aureus* and *S. agalactiae* (11 ± 0.3 and 12 ± 0.6 mm inhibition zones). Likewise, the percentage of peripheral blood mononuclear cells (PBMC) viability was increased in response to MM, IC_50_ values (1.781 ± 1.2 and 6.545 ± 0.93 µg/mL) for ethanol and aqueous extracts, respectively. In addition, our results showed that the MM extract is safe even at a high dose of 5,000 mg/kg and has no oral toxicity. These findings suggest the excellent medicinal bioactivity of MM and explain the popularity of this plant in the folk medicine as a remedy for different illnesses.

## 1. Introduction

Plants derived natural products are the source of most active components of medications, which in turn play a significant role in the treatment or prevention of human illnesses. Tropical plants have been investigated intensively during the last decades in order to evaluate the possibility of developing new, sustainable, natural and affordable cosmetics and drugs [1]. *Melastoma malabathricum* (MM) is a plant from the family (Melastomataceae) found in many regions of tropical Asia where it is known by different local names such as the Straits Rhododendron (Singapore), Malabar melastome (Australia), Indian-rhododendron or Lutki (India) and Senduduk (Malaysia). It has been used in the traditional medicine to treat diarrhea, accelerate wound healing, lowering high blood pressure, treat diabetes, prevent scarring of smallpox and to treat piles [2,3]. The most recent research on MM revealed that its bioactive constituents exhibited free radical scavenging activity and anti-inflammatory effects on mouse ear edema [4], gastroprotective effects against ethanol-induced gastric ulcers in rats [5], antibacterial effects against different strains of bacteria [6,7,8], effective anticoagulant activity [9], it exhibited antiviral and cytotoxic effect against murine cell lines [10], and possesses antinociceptive, anti-inflammatory, and antipyretic activities [11,12] and the natural flavonoid and pentacyclic triterpenes isolated from it possess anti-inflammatory effects [13].

In addition, Faravani [14] reported the antioxidant activity of crude and methanol extracts of *M. Malabathricum* using the DPPH assay. The antioxidant potential of white petals *M. Malabathricum* was determined by Susanti *et al*. [4] using the ferric thiocyanate (FTC) and DPPH (UV and ESR spectroscopic) methods.

On the basis of its traditional use and literature reference, this study was designated to evaluate *Melastoma malabathricum’s* oral acute toxicity on Sprague Dawley rats *in vivo* and scavenging activity toward DPPH, ABTS and FRAP free radicals, phytochemical screening of total phenolic and flavonoids contents, antibacterial effects against *Staphylococcus aureus*, *Streptococcus agalactiae*, *Escherichia coli and Klebsilla pneumonia* and immunomodulatory role on human peripheral blood mononuclear cell (PBMC) *in vitro*.

## 2. Results and Discussion

### 2.1. Antioxidant Activity

Nowadays, naturally occurring antioxidants are mostly preferred due to their little or no toxicity in comparison with some synthetic antioxidants which have been documented to have toxic or mutagenic effects. Polyphenols are the largest group of antioxidants that have the ability to up-regulate some metal chelating reactions and scavenge free radicals, like singlet oxygen and hydrogen peroxide which should be regularly removed from cells to maintain their normal functions [15]. Flavonoids are the main group of polyphenols that have been publicized to act as scavengers of different oxidizing species like hydroxyl radical, superoxide anion or peroxy radicals [16]. One of the undoubted roles of flavonoids is their function in protecting plants against microbial attack and accumulation as phytoalexins in response to microbial attack. Moreover; some flavonoids has been documented recently to exhibit antibacterial activity against different strains of bacteria.

Table 1 shows that the aqueous extract of MM showed better activity toward DPPH radicals since it showed a lower IC_50_ value (10.573 ± 0.58 µmol/L) than the ethanol extract (11.599 ± 0.84 µmol/L) while interestingly, the ethanol extract demonstrated better ABTS free radicals scavenging activities (62.657 ± 0.78 µmol/L) than the aqueous extract (63.939 ± 0.48 µmol/L) and the ethanol extract also showed relatively strong ferric reducing antioxidant power (33,590 ± 0.038 mmol/g). These findings confirm the findings of Faravani [14] and Susanti *et al*. [4] who reported the ability of *M. malabathricum* extracts to scavenge the DPPH free radicals. Also Zakaria *et al*. [17] determined that the aqueous and methanol extracts of MM leaves had high antioxidant activity in the superoxide and DPPH scavenging assays and they recorded that the antiproliferative effects of MM could be attributed to its high content of phenolic compounds.

**Table 1:** DPPH, ABTS, FRAP radical scavenging activities with total phenolic and total flavonoids contents of MM*.*

	Vitamin C	BHT	MM/A	MM/E
DPPH (IC_50_ µmol/L)	3.346 ± 1.20	…	10.573 ± 0.58	11.599 ± 0.84
ABTS (IC_50_ µmol/L)	21.368 ± 0.12	…	63.939 ± 0.48	62.657 ± 0.78
TPC (mg/g)	…	…	379.33 ± 0.007	384.33 ± 0.005
TF (mg/g)	…	…	14.7 ± 0.003	85.8 ± 0.009
FRAP (mmol/g)	…	57,300 ± 0.01	28,750 ± 0.031	33,590 ± 0.038

Values are represented as mean ± SEM for triplicates; BHT = butylated hydroxytoluene; MM/A = *M. malabathricum* aqueous extract; MM/E = *M. malabathricum* ethanol extract.

Moreover, phytochemical screening of MM revealed that the ethanol extract showed higher total phenolics (384.33 ± 0.005 mg/g) and flavonoids contents (85.8 ± 0.009 mg/g). These results revealed that the free radical scavenging activity might not be related to the presence of phenols components, These findings support the previous research of [18] who reported that *Cassia tora* extract possesses potent antiradical and antioxidant which is not correlated with the total phenols content of the same plant. However, our findings disagree with findings of Chalise *et al*. [19] who evidenced that methanol and water extracts of MM fruits showed correlation between DPPH free radical scavenging activity and total phenolic contents in the plant.

### 2.2. Antibacterial Activity

Ethanol and aqueous extracts of MM displayed antibacterial activity against Gram positive bacteria only: *Staphylococcus aureus* (11 mm and 6 mm) and *Streptococcus agalactiae* (6 mm and 12 mm) of inhibition zones respectively, as shown in Table 2, while there were no activities against the Gram negative bacteria *Escherichia coli* and *Klebsilla pneumonia*.

**Table 2:** MM’s antibacterial activity measured by the disc diffusion method.

Bacterial species	CTRL	DMSO	*M. malabathricum*	
			AE	EE
*E. coli*	27 ± 1.4	NIZ	NIZ	NIZ
*K. pneumonia*	31 ± 0.6	NIZ	NIZ	NIZ
*S. agalactiae*	16 ± 0.6	NIZ	6 ± 0.2	12 ± 0.6
*S. aureus*	12 ± 0.3	NIZ	6 ± 0.1	11 ± 0.3

Values are represented as mean inhibition zone (mm) ± SEM of triplicates; CTRL = the antibiotic positive control; NIZ = no inhibition zone; AE = aqueous extract; EE = Ethanol extract.

Both *Staphylococcus aureus* and *Streptococcus agalactiae* had minimal inhibitory concentration (MIC) values (1.25 µg/mL) for the ethanol extract of MM and the minimal bactericidal concentration (MBC) values were next to MIC values (2.5 µg/mL), as shown in Table 3.

**Table 3:** Minimal inhibitory concentrations (MIC) and Minimal bactericidal conentrations (MBC) of MM’s ethanol extract against *S. agalactiae* and *S. aureus*.

MM concentration	MIC / *S.agalactiae*	MBC / *S.agalactiae*	MIC / *S.aureus*	MBC / *S.aureus*
20 µg/mL	No turbidity	No growth	No turbidity	No growth
10 µg/mL	No turbidity	No growth	No turbidity	No growth
5 µg/mL	No turbidity	No growth	No turbidity	No growth
2.5 µg/mL	No turbidity	**No growth**	No turbidity	**No growth**
1.25 µg/mL	**No turbidity**	Growth	**No turbidity**	Growth
0.625 µg/mL	Turbid	Growth	Turbid	Growth
0.3125 µg/mL	Turbid	Growth	Turbid	Growth
0.1563 µg/mL	Turbid	Growth	Turbid	Growth

Results of MM’s antibacterial activity at a concentration 200 mg/mL of aqueous and ethanolic extracts showed no inhibitory effect against the growth of Gram negative bacteria but they showed very clear activity against Gram positive bacteria, as shown in Table 2. The fact that Gram-positive bacteria were more sensitive to the antimicrobial compounds in herbs and medicinal plants than Gram-negative bacteria is due to the presence of lipopolysaccharides (LPS) in the Gram negative bacteria’s cell wall, which form a zone outside its cytoplasmic membrane and organize the passage of molecules through it. Accordingly, chemicals face difficulties in penetrating these membranes and, consequently, their effectiveness is restricted. For that reason, aqueous and ethanolic extracts of MM showed antibacterial activity against *Staphylococcus aureus* and *Streptococcus agalactiae*, but not against *Escherichia* *coli* and *Klebsilla pneumonia*. The antibacterial effect of MM is due to the high levels of phenols and flavonoids that have been reported as major antimicrobial component in medicinal plants [20] probably due to their capability to interact with extracellular soluble proteins and to interact with bacterial cell walls; in addition some lipophilic flavonoids can disrupt microbial cell membranes. These findings are in consistent with those of the other studies of Choudhury *et al*. [6] who evaluated the antibacterial activity of methanol and acetone extracts of *M. malabathricum* against *Staphylococcus aureus*, *Streptococcus* sp. and *E. coli* by the disc diffusion method. Their findings showed significant zones of inhibitions against all tested bacteria and according to Sunilson *et al*. [8] the methanol extract of MM exhibited antibacterial effects against different clinical wound isolates of *S*. *aureus* and *P*. *aeruginosa*. Additionally, Wang *et al*. [7] studied the antibacterial activity of water, acetone, ethanol and ethyl acetate extracts of *Melastoma candidum* and they found that *Melastoma candidum* exhibited a good antibacterial activity, especially the acetone extract. Moreover, Wong *et al*. [21] reported the isolation of 12 compounds from MM, some of them possessing antibacterial activity and also [22] studied the antibacterial effects of MM and showed that this plant demonstrated antibacterial activity against *B. subtilis*, *S. aureus*, *E. coli*, and *P. aeruginosa* with MIC value ranging between 62.5 and 125.0 μg/mL.

### 2.3. PBMC Proliferation

**Figure 1:**
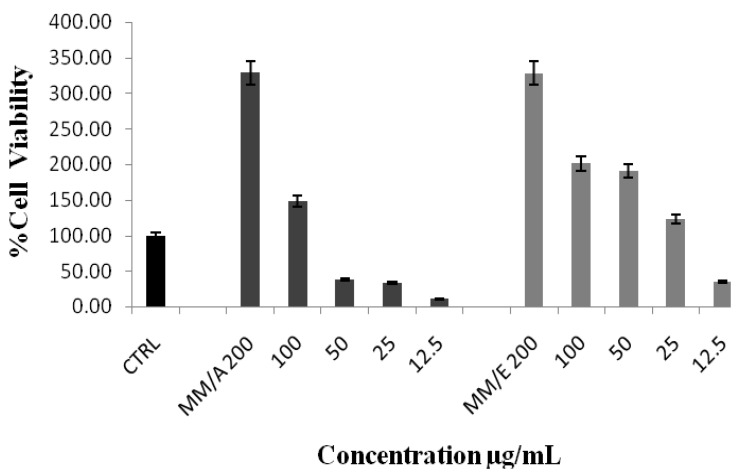
MM’s immunomodulatory effect on human PBMC proliferation, CTRL = distilled water, MM/A = *M. malabathricum* aqueous extract, MM/E = *M. malabathricum* ethanol extract.

Our immunomodulatory study on the effects of MM on human peripheral blood mononuclear cells (PBMC) evidenced that both this plant’s extracts exhibits a strong ability to proliferate the viability of PBMC, as shown in Figure 1. IC_50_ values were (1.781 ± 1.2) and (6.545 ± 0.93) µg/mL for ethanol and aqueous extracts, respectively.

The 3-(4,5-dimethylthiazol-2-yl)-2‚5-diphenyltetrazolium bromide (MTT) colorimetric assay is one of the most commonly used techniques for determining cytotoxicity and cell proliferation. The MTT is reduced by active mitochondria to a dark purple, coloured insoluble formazan product which can be measured spectrophotometrically and considered as the measure of the viability of the cells since the reduction of MTT occurs only in metabolically active cells [23]. Our results demonstrate that both extracts induced the proliferation of white blood cells after 24 h of incubation which suggests that our extracts are not toxic to normal immune cells and therefore exhibits a potential to modulate the cellular immune system. The excellent immunomodulatory activity of MM in the present study may be due to the presence of quercetin, which was identified previously as one of its active components [4]. These findings seem to be consistent with other research of Nair *et al*. [24] that the flavonoid quercetin has the ability to modulate the immune response and increase the percentage of peripheral blood mononuclear cells.

### 2.4. Acute Toxicity Test

**Figure 2:**
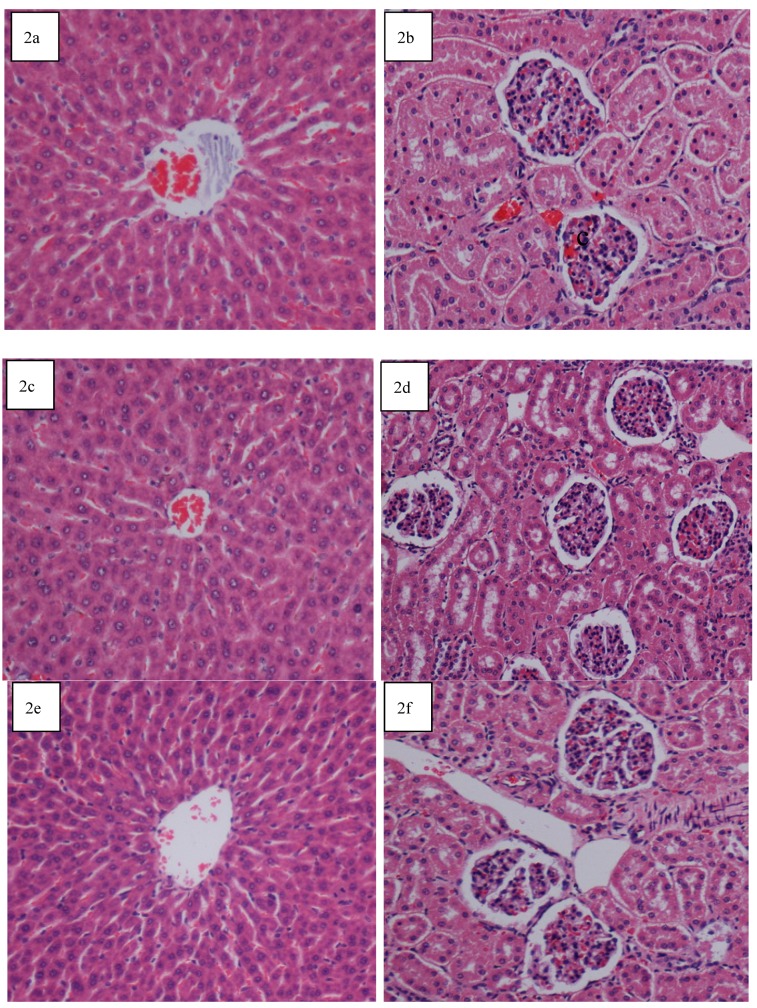
Histological sections of liver and kidney from the acute toxicity test. (**2a** and **2b**) Control treated with 5 mL/kg vehicle (10% Tween 20); (**2c** and **2d**) MM extract (2,000 mg/kg); (**2e** and **2f**) MM extract (5,000 mg/kg). No structural differences were seen between the MM groups and control group. (H & E stain; 20× magnification).

The acute toxicity study at doses of 2,000 mg/kg and 5,000 mg/kg did not result in any mortality in the treated rats and no toxic effects were observed throughout the 14 days study period. Physical observations indicated no signs of changes in rats’ skin and fur, eyes and mucus membrane, behavior patterns, tremors, salivation, diarrhea and sleep. The body weight of the treated male and female rats increased gradually but were not significantly different as compared to those of the control rats. Gross necropsy findings did not reveal any changes in the organs. The clinical observations, biochemical measurements reflected normal status of the kidney and liver functions, and histopathological evaluations of these organs all together revealed that there were no significant differences between the control and the test groups, as shown by the quantitative data in Table 4 and Table 5 and qualitative data in Figure 2. The findings from the application of the MM extract to the control rats provided sufficient evidence to conclude that the orally administered extract was safe and presented no extract-related toxicity even at the highest dose of 5,000 mg/kg.

**Table 4:** Effects 2,000 mg/kg and 5,000 mg/kg of MM extract on liver biochemical parameters.

Groups	ALT (IU/L)	AST (IU/L)	ALP (IU/L)	T. Protein (g/L)	Albumin (g/L)	Bilirubin (µmol/L)	GG (IU/L)
Tween 20 (10%)	45.17 ± 5.26	27.33 ± 4.22	98.67 ± 13.12	73.33 ± 3.14	42.67 ± 3.04	10.67 ± 2.16	53.83 ± 10.42
MM extract (2,000 mg/kg)	46.50 ± 4.21	26.83 ± 3.84	92.83 ± 11.97	72.67 ± 2.09	45.67 ± 6.73	6.67 ± 1.41	55.00 ± 10.58
MM extract (5,000 mg/kg)	44.33 ± 5.94	28.00 ± 3.84	88.17 ± 6.58	74.33 ± 4.62	44.17 ± 4.05	9.50 ± 1.65	58.83 ± 7.15

Values were expressed as mean ± S.E.M. There are no statistically significant differences between the measurements in different groups. The significant value was set at *p* < 0.05.

**Table 5:** Effects 2,000 mg/kg and 5,000 mg/kg of MM extract on kidney biochemical parameters.

Groups	Sodium	Potassium	Chloride	Carbon dioxide	Anion Gap	Urea	Creatinine
Tween 20 (10%)	140.50 ± 1.87	4.30 ± 0.28	104.33 ± 1.69	26.00 ± 1.67	14.50 ± 1.67	4.60 ± 0.71	37.25 ± 0.15
MM extract (2,000 mg/kg)	140.33 ± 1.20	4.54 ± 0.23	105.33 ± 2.08	27.33 ± 1.50	12.80 ± 1.33	4.62 ± 0.65	37.48 ± 0.16
MM extract (5,000 mg/kg)	136.33 ± 1.50	4.28 ± 2.25	104.60 ± 1.54	25.50 ± 1.15	19.00 ± 0.52	4.56 ± 0.56	36.58 ± 0.82

Values were expressed as mean ± S.E.M. There are no statistically significant differences between the measurements in different groups. The significant value was set at *p* < 0.05.

## 3. Experimental

### 3.1. Plant Extraction Procedure

MM was obtained from Ethno Resources Sdn Bhd, Selangor Malaysia. For preparation of ethanol extract 100 g was soaked in 1,000 mL of 95% ethanol for three days then the mixture was filtered by filter paper (Whatman No. 1, Fitchburg, WI, USA) and extracted under compact pressure in a rotating evaporator (Buchi, Flawil, Switzerland). While, the aqueous extracts were prepared by soaking 100 g of MM in 2,000 mL of distilled water then shaking for four hours in a water bath; the filtered mixture wascstored inside freezer to make small ice blocks and the plant extract was obtained by using a freeze drying machine (Labconco, Kansas City, MO, USA), All extracts were kept at −20 °C until the tests were performed. 

### 3.2. Free Radical Scavenging Activity

#### 3.2.1. DPPH Method

The method of Lim and Murtijaya [25] was used for the DPPH assay with slight modifications. The scavenging action of stable 2,2-diphenyl-1-picryl dyhydrazyl free radical was determined. Five µL of samples/standards were loaded, and followed by 195 µL of DPPH reagent, the mixtures were then mixed vigorously and incubated at room temperature in the dark for 2 h and the absorbance was measured spectrophotometrically at 515 nm. 

#### 3.2.2. ABTS Method

The ABTS assay was performed using the method of Re *et al*. [26]; briefly, 7 mM ABTS and 2.45 mM potassium persulfate reacted and were kept at room temperature, in a dark place for 12–16 h. This solution was diluted to an absorbance of (0.70 ± 0.02) at 734 nm and equilibrated at 30 °C while distilled water was used to dilute the plant extracts. To each 1 mL of prepared ABTS, we added 10 µL of each plant extract and mixed them carefully. The mixtures were allowed to sit for 15 min at room temperature and the absorbance was read at 734 nm. 

#### 3.2.3. FRAP Method

The ferric reducing activity (FRAP) of the plant extracts were estimated using the method developed by Benzie and Strain [27]. The reaction mixture contained 300 mmol/L acetate buffer, 10 mmol/L 2,4,6-tripyridyl-*s*-triazine (TPTZ) in 40 mmol/L of HCL and 20 mmol/L of FeCl_3_·6H_2_O; the working FRAP reagent was prepared freshly by mixing 25 mL of acetate buffer, 2.5 mL of TPTZ solution and 2.5 mL of FeCl_3_·6H_2_O. The freshly prepared mixture was incubated at 37 °C in a water bath for five minutes and then a blank reading was taken spectrophotometrically at 593 nm. After that, 30 µL of extract or standard and 90 µL of distilled water were added to 900 µL of the working FRAP reagent. Absorbance was measured at 0 min immediately upon addition of the working FRAP reagent after vortexing. Thereafter, absorbance reading was taken after four minutes.

### 3.3. Phytochemical Screening

#### 3.3.1. TPC Method

Total phenolic content of the plants were determined using Folin-Ciocalteu reagent using gallic acid as a standard. Ten µL of extract solution (1 mg/mL) was added, followed by 0.5 mL of 1:10 Folin-Ciocalteu reagent. The mixture was incubated at room temperature for 5 min then 0.35 mL of 115 mg/mL natrium carbonate (Na_2_CO_3_) was added and mixed thoroughly. The mixture was then placed for 2 h at room temperature. Absorbance readings were taken spectrophotometrically at 765 nm. 

#### 3.3.2. TFC Method

Total flavonoids content was determined by aluminium chloride colorimetric method by Chang *et al*. [28]. Briefly, 0.5 mL of extract solutions (1 mg/1 mL) were added to a separate test tube and mixed with 1.5 mL of 0.95 ethanol, 0.1 mL of 1 M of potassium acetate, 0.1 mL of aluminium chloride and 2.8 mL of distilled water. The mixtures were incubated for 30 min at room temperature. The absorbance readings were taken spectrophotometrically at 415 nm.

### 3.4. Antibacterial Activity

#### 3.4.1. Bacterial Strains and Antibiotics

*In vitro* antibacterial activity was examined for aqueous and ethanol extracts of MM, the following bacterial strains and their reference antibiotics were employed in the tests: *Escherichia coli* ATCC 25922 (Gentamycin 30 µg), *Klebsilla pneumonia* ATCC 1937000 (gentamycin 30 µg), *Staphylococcus aureus* ATCC 25923 (vancomycin 5 µg) and *Streptococcus agalactiae* were laboratory isolates obtained from the Molecular Bacteriology Laboratory/Molecular Medicine Department / Faculty of Medicine / University of Malaya.

#### 3.4.2. Disc Diffusion Method

The tests were performed using Muller Hinton agar for bacterial strains using the Kirby-Bauer’s disc diffusion method following the National Committee for Clinical Laboratory Standards methods [29]. The sterile petri dishes containing solid and sterile Muller Hinton agar were used. Sterile (6 mm) paper disc (Oxoid, Hampshire, UK) was saturated with 50 µL of sample at a concentration of 5 mg/disc dissolved in DMSO. The bacterial suspension which was prepared from a 24 h culture was adjusted to an inoculation of 1 × 10^7^ cfu/mL. Turbidity at 600 nm was adjusted with sterile broth media. The dried surface of the Muller Hinton agar plate was streaked, and five dried discs were placed per petri dish including the negative control (DMSO) and the commercially available antibiotic. The plates were then incubated at 37 *°*C for 18–24 h. Microbial growth was indicated by measuring the diameter of the zone of inhibition.

#### 3.4.3. MIC and MBC Methods

The minimal inhibitory concentration (MIC) and minimal bactericidal concentration (MBC) methods were applicable to the extracts that established their effectiveness to bacteria by the disk diffusion method (inhibition zone ≥ 8 mm). Each extract was issued to eight serial dilutions by using sterile Muller-Hinton broth (20, 10, 5, 2.5, 1.25, 0.625, 0.3125 and 0.1563 µg/mL), then 10 μL of bacteria (in the log phase) was inoculated to each dilution separately and incubated at 37 °C for 18–24 h. The higher dilution of the plant extracts that showed an absence of turbidity of bacteria is the MIC of the extract. The minimal bactericidal concentration (MBC) was identified as the lower concentration of the plant to kill the microorganisms. It established by culturing the sample dilution on a fresh Muller-Hinton agar medium and incubating further at 37 °C for 18–24 h.

### 3.5. PBMC Proliferation Activity

Ten milliliters of whole blood was drawn from a healthy donor and diluted with the same volume of Histopaque. The mixed solution was centrifuged under at 1,000 rpm for 30 min. The mononuclear layer was carefully transferred out and washed, then pelleted down with 30 mL PBS (phosphate buffer saline) and centrifuged at 1,000 rpm for 10 min for thrice and re-suspended with RPMI media supplemented with 2 mM glutamine and NaHCO_3_ (Sigma-Aldrich, Gillingham, UK) with addition of 10% FBS (Fetal Bovine Serum). Counting of cells was done by Neubaur haemocytometer (Weber, Teddington, UK) to find out the PBMC cell number with the same volume of trypan blue. MTT (Merck, Darmstadt, Germany) assay was used to study the effect of the extract on cell viability. Briefly, 100 μL of cells suspended in RPMI media (with 10% of FBS) was added into the 96 well plate and incubated at IR Jacketed incubator (from NUAIRE laboratory equipment supply, Plymouth, MN, USA) for 24 h at 37 °C. Then, 10 μL of the extracts (20 mg/mL) were added and further incubated for 24 h. After the corresponding period 10 µL of MTT reagent (5 mg/mL PBS) was added into each well and further incubated for 4 h. 100 µL of DMSO were added and shake for 20 min to solubilize and extract the formazan crystal. At the last, the plate was read at 595 nm by using Power wave X 340 ELISA plate reader (from BIO-TEK instruments, Winooski, VT, USA). All extract samples and controls were tested in triplicates in four independent experiments. The percentage of cell viability was calculated by the formula: 


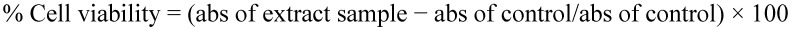


### 3.6. Acute Toxicity Test

To establish the safety of the MM extract when administered orally, the acute toxicity test was evaluated following OECD-423 guidelines [30]. This study was approved by the institutional Ethics Committee, University of Malaya, Malaysia with protocol number PM/07/05/2011/1111/MAA (a) (R). Throughout the experiments, we provided human care to the animals according to the criteria outlined in the “Guide for the Care and Use of laboratory Animals” prepared by the National Academy of Sciences and published by the National Institutes of Health. Adult male healthy Sprague Dawley rats weighing between 190–260 g were acquired from the experimental animal house in our institute. The rats were placed individually in a separate cage and maintained on standard pellet diet and tap water. Twenty four healthy Sprague Dawley rats (12 males and 12 females) were randomly assigned equally into 3 groups labeled as vehicle 10% Tween 20 and two doses 2,000 mg/kg and 5,000 mg/kg of the extract, respectively. Each rat was made to fast (no food but water) overnight prior to dosing. Food was withheld for another 3 to 4 h after the dosing. The rats were closely observed for 30 min and at 2, 4, 24 and 48 h after the dosing to detect if there were any acute signs of clinical or toxicological symptoms. After 14 days the rats were sacrificed to measure serum biochemical and (liver and kidney) histological parameters by following the standard methods [31]. Blood of each rat was collected in gel activated clot tubes and serum was collected for analysis in the Clinical Diagnostic Laboratory (CDL) at University of Malaya Medical Center (UMMC) to determine the liver function enzymes such as alanine aminotransferase (ALT), aspartate aminotransferase (AST), alkaline phosphatase (ALP), Bilirubin, albumin, total protein (TP) and gamma glutamyl transferase (GG). A small liver and kidney specimens from all the experimental groups were subsequently fixed in 10% buffered formalin and embedded in paraffin using automated tissue processing machine (Leica, Nussloch, Germany). Sections of the liver were sliced at 5 µm thickness and stained with haematoxylin and eosin (H&E) for histological evaluation.

## 4. Conclusions

Based on the findings of this study we can conclude that MM is a biologically active plant and that explains the popularity of this plant in the folk medicine as a remedy for different illnesses.
